# Identification of the core region responsible for the activity of *CmVg* promoter and its regulatory transcription factor BrC in *Cnaphalocrocis medinalis* (Lepidoptera: Pyralidae)

**DOI:** 10.1093/jisesa/ieag057

**Published:** 2026-07-21

**Authors:** Lingling Long, Dong Jin, Hongshuai Gao, Qiao Gao, Wenbing Ding, Lin Qiu, Youzhi Li, Hualiang He

**Affiliations:** Hunan Provincial Key Laboratory for Biology and Control of Plant Diseases and Insect Pests, College of Plant Protection, Hunan Agricultural University, Changsha, China; Hunan Provincial Key Laboratory for Biology and Control of Plant Diseases and Insect Pests, College of Plant Protection, Hunan Agricultural University, Changsha, China; Hunan Provincial Key Laboratory for Biology and Control of Plant Diseases and Insect Pests, College of Plant Protection, Hunan Agricultural University, Changsha, China; Hunan Provincial Key Laboratory for Biology and Control of Plant Diseases and Insect Pests, College of Plant Protection, Hunan Agricultural University, Changsha, China; Hunan Provincial Key Laboratory for Biology and Control of Plant Diseases and Insect Pests, College of Plant Protection, Hunan Agricultural University, Changsha, China; Hunan Provincial Key Laboratory for Biology and Control of Plant Diseases and Insect Pests, College of Plant Protection, Hunan Agricultural University, Changsha, China; Hunan Provincial Key Laboratory for Biology and Control of Plant Diseases and Insect Pests, College of Plant Protection, Hunan Agricultural University, Changsha, China; Hunan Provincial Key Laboratory for Biology and Control of Plant Diseases and Insect Pests, College of Plant Protection, Hunan Agricultural University, Changsha, China

**Keywords:** vitellogenin, promoter, Broad‑Complex, transcriptional regulation, *Cnaphalocrocis medinalis*

## Abstract

Insect reproduction depends critically on vitellogenesis, during which vitellogenin (*Vg*) is synthesized in the female fat body and incorporated into developing oocytes as an essential nutrient source. In this study, the promoter region of the vitellogenin gene (*CmVg*) of the rice leaf folder (*Cnaphalocrocis medinalis*) was predicted and cloned. A core active region was delineated within the *CmVg* promoter, and bioinformatic analysis revealed several predicted cis‑regulatory elements (CREs) for Broad‑Complex (BrC) transcription factors in the core region. Among four BrC protein candidates in the genome of *C. medinalis*, *Cmed074000.1* significantly enhanced the transcriptional activity of *CmVg*. Disruption of the CRE of *Cmed074000.1* strongly reduced the expression level of the reporter. Moreover, RNA interference-mediated knockdown of *Cmed074000.1* in adult females significantly down‑regulated *CmVg* transcriptional levels and severely impaired egg production by 62.24%. This work provides the first evidence of a transcriptional pathway controlling *CmVg* expression in this pest, *C. medinalis*, offering novel insights into its reproductive biology and highlighting a potential target for future pest‑management strategies.

## Graphical abstract

**Graphical Abstract:**
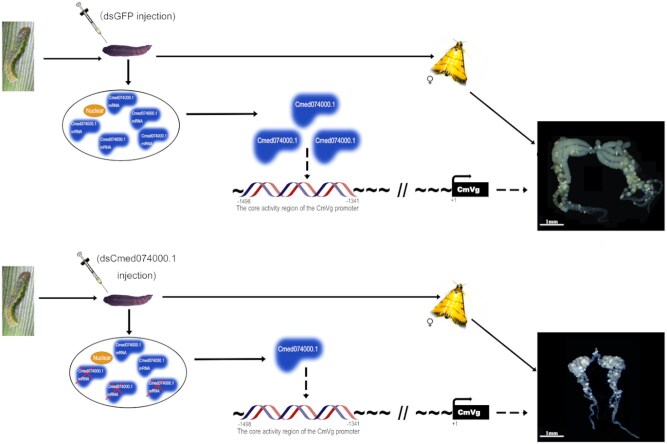

## Introduction

In most insects, vitellogenin (Vg) functions as a critical female‑specific lipoprotein synthesized, transported, and ultimately deposited into developing oocytes under the control of ovarian signaling pathways. This precursor is ultimately converted to vitellin (Vt), the predominant yolk‑protein storage form within the egg, thereby establishing a vital molecular link between maternal reproductive physiology and embryonic nutritional supply (Valle 1993, Yang et al. 2014a). The reproductive output of oviparous insects is therefore directly contingent upon the efficacy of *Vg* uptake during oocyte vitellogenesis. *Vg* is widely distributed in the hemolymph, fat body, and eggs of oviparous vertebrates and invertebrates (Tufail and Takeda 2008). As a central regulatory process in reproduction, vitellogenesis has been extensively investigated (Tomoyasu and Denell 2004, Jairin et al. 2013), and *Vg* itself is frequently employed as a molecular marker for assessing reproductive status and sex identification.

In insects, key physiological processes such as reproduction are orchestrated by the coordinated actions of ecdysone, juvenile hormone, and other neuropeptides. Among these, the ecdysone signaling pathway plays a particularly critical role (Leyria 2024, Li et al. 2024). This pathway is mediated by a heterodimeric nuclear receptor complex composed of the Ecdysone Receptor (*EcR*) and its partner Ultraspiracle (*USP*), which initiates a transcriptional cascade by activating primary-response early genes, followed by secondary-response late genes, thereby precisely regulating the expression of downstream target genes. A notable example is the synthesis of *Vg*, whose transcription is directly governed by this hormonal pathway: binding sites for the ecdysteroid receptor complex (*EcR-USP*) and early-response transcription factors such as *BrC, E74*, and *E75* in *Aedes aegypti* (Zhu et al. 2007). This provides molecular evidence for how hormones precisely regulate *Vg* gene expression through specific transcription factors.

The Broad-Complex (*BrC*), a critical early-response gene in the ecdysone signaling cascade, encodes a family of DNA-binding proteins characterized by an N-terminal BTB domain and C2H2-type zinc finger domains (Zollman et al. 1994, Waltzer et al. 2016). These domains are highly conserved across diverse insect species (Nishita and Takiya 2004). In *Drosophila melanogaster*, alternative splicing generates four major isoforms (Z1-Z4) that share the common BTB domain but are distinguished by their unique C2H2 zinc finger motifs (DiBello et al. 1991). Subsequent investigations have revealed multiple BrC isoforms (eg Z1-Z4, Z1-Z6) in various insect species, including *Manduca sexta* (Zhou et al. 1998), *Apis mellifera* (Paul et al. 2006), *Blattella germanica* (Piulachs et al. 2010), *Psacothea hilaris* (Nagamine et al. 2014), and *Bombyx mori* (Yang et al. 2014b). Notably, isoforms such as Z1, Z2, and Z4 in *B. mori* exhibit functional conservation with those in *Drosophila* (Reza et al. 2004, Lin et al. 2017, Zhen et al. 2018). Additionally, isoforms lacking a zinc finger domain have been reported within the same species. Functionally, BrC regulates a wide array of downstream genes and is essential for developmental processes such as metamorphosis and pupal differentiation, as evidenced by studies of *Drosophila* mutants (Zhou et al. 1998, Paul et al. 2006) and RNAi experiments in insects like *Oncopeltus fasciatus*, *Tribolium castaneum*, *Chrysopa perla*, and *B. mori* (Ijiro et al. 2004, Piulachs et al. 2010, Nagamine et al. 2014). Beyond development, BrC plays a role in reproductive regulation, as demonstrated in the German cockroach *B. germanica* and the mosquito *A. aegypti* (Chen et al. 2004). Notably, in *A. aegypti*, specific isoforms exert distinct regulatory effects on vitellogenin expression, with BrC-Z1 and Z4 acting as repressors and Z2 enhancing the 20E-mediated activation of the *Vg* promoter via the ecdysone receptor (*EcR-USP*) (Chen et al. 2004, Zhu et al. 2007, Zhen et al. 2018).

The rice leaf folder, *Cnaphalocrocis medinalis*, is a long‑distance migratory insect whose larvae feed by rolling and consuming rice leaves, rendering it a persistent and economically important pest of rice across Asia (Li et al. 2022, Wang et al. 2025). This species exhibits strong reproductive capacity, with each female adult capable of laying 40 to 50 eggs in a scattered manner over its lifespan, and in some cases, more than 200 eggs (Sun et al. 2013, Fu et al. 2014). Moreover, exposure to non‑target pesticides such as imidacloprid and buprofezin, commonly applied for planthopper control, can markedly enhance the oviposition rate of *C. medinalis*, a factor that contributes substantially to its population resurgence (Chintalapati et al. 2019, Mateos Fernandez et al. 2022, Yang et al. 2025). Consequently, elucidating the development of its eggs and ovaries, along with the diverse factors that govern fecundity, is of critical importance for designing effective management strategies against this pest. To date, limited research has been conducted on the molecular basis of its reproductive capacity and regulatory mechanisms of *Vg* in *C.medinalis*. In this study, we investigated the promoter activity of the *Vg* gene in *C. medinalis* and identified transcription factors that critically regulate *Vg* transcription.

## Materials and Methods

### Insect and Cell Culture

The laboratory strain of *C. medinalis* was established from egg masses obtained from the Hunan Academy of Agricultural Sciences in 2024 (Changsha, China) and maintained without insecticide exposure. All experiments used third‑generation insects reared on wheat seedlings (Zhu et al. 2015) to minimize epigenetic effects. Larvae were reared on wheat seedlings under controlled environmental conditions (27 ± 1 °C, 70% to 80% RH, and a 16:8 h L:D photoperiod).

HEK293T cells were maintained in high-glucose Dulbecco’s Modified Eagle Medium (DMEM; Pricella) supplemented with 10% fetal bovine serum (FBS) at 37 °C in a humidified 5% CO_2_ atmosphere. Cells were routinely subcultured every 2 to 3 days at a split ratio of 1:5 to 1:10 upon reaching approximately 75% confluence.

### Genomic DNA Extraction and cDNA Synthesis

Genomic DNA was extracted from adult female *C. medinalis* using the Ezup Column Animal Genomic DNA Purification Kit (Sangon Biotech, Shanghai, China). DNA concentration and purity were assessed using a NanoDrop 1000 spectrophotometer (Thermo Scientific, Waltham, MA, USA). Purity was evaluated based on the A_260_/A_280_ ratio. The DNA was then diluted to a working concentration of 50 ng/μL for subsequent analysis. Total RNA was extracted from the samples using Trizol reagent (Vazyme, Nanjing, China).cDNA was synthesized from 1 μg of total RNA using the Hifair III First Strand cDNA Synthesis Kit (YEASEN, Shanghai, China). This kit includes a genomic DNA removal step.

### Sequence Analysis of *CmVg* Promoter

Based on the sequence of the *C. medinalis CmVg* gene published in NCBI, we intercepted the region within 2000 bp upstream of the start-codon of translation initiation of the *CmVg* gene as the proposed promoter region of the *CmVg* gene. Then, the online software (http://jaspar.genereg.net/) was used to predict and select the potential transcription factor binding sites of *CmVg*. Based on the prediction results, six specific primers of truncated fragments of different lengths were designed using Primer premier 5.0 software (Supplementary Table S1). All primers used in this study were synthesized by Qingke Zixi Biotechnology Co., Ltd., Changsha, China. We named the six truncated fragments F1 (−386 to −1), F2 (−718 to −1), F3 (−1123 to −1), F4 (−1566 to −1), F5 (−1921 to −1), and F6 (−2306 to −1).

### Vector Construction and Transfection

The six truncated fragments of the *CmVg* promoter were individually cloned into the pGL4.10-Basic vector (Promega, USA) using *Hind*III and *Xho*I restriction sites. Following digestion, fragments were ligated into the linearized vector using T4 DNA ligase (TaKaRa, Japan). The resulting plasmid constructs were subsequently transfected into HEK-293T human embryonic kidney cells.

HEK293T cells at 85% confluence were co-transfected with the recombinant plasmids (F1 to F6, F4-1, F4-2, F4-3, F4-4) and the pGL-4.73 vector using Lipofectamine 3000 (Invitrogen, USA). In parallel, cells were co-transfected with the basic empty vector and pGL-4.73 as a negative control. Forty-eight hours post-transfection, cells were washed with PBS and lysed with 200 μL Passive Lysis Buffer (PLB) followed by gentle rocking for 30 min at room temperature. Luciferase activity was measured using the Dual-Luciferase Reporter Assay System (Promega).

### Transcription Factor Binding Site Prediction

The recombinant plasmid pGL‑158bp was generated by cloning the 158 bp core active region of the *CmVg* promoter into the pGL4.10-Basic vector. To functionally dissect the promoter, transcription factor binding sites (TFBS) were predicted via bioinformatic analysis of the JASPAR 2024 database (relative profile score threshold: 90%). This analysis identified two potential binding sites for the BrC factor, located at positions −1431 to −1445 and −1396 to −1407. Using site-directed mutagenesis, mutant promoter constructs were generated by independently disrupting each BrC site. The functional impact of these mutations was then assessed by comparing the transcriptional activity of each mutant plasmid to the wild-type promoter in a dual-luciferase reporter assay.

### RNAi Assay

Double-stranded RNAs (dsRNAs) were synthesized and microinjected according to established methods (Lin et al. 2013). A target region corresponding to a specific domain at the C-terminus of *Cmed074000.1* (amino acid positions 128 to 403) was selected for dsRNA synthesis. This fragment was PCR-amplified with flanking T7 promoter sequences and cloned into a blunt-ended vector. dsRNAs were synthesized in vitro using the T7 RiboMAX Express RNAi System (Promega, Madison, WI, USA), with dsRNA targeting the *GFP* gene serving as a negative control. All primer sequences are listed in Supplementary Table S1.

Female pupae were used for microinjection. Injections were performed on day‑6 pupae (ie 6 days post‑pupation, approximately 24 h prior to expected adult eclosion). Late-pupal-stage female *C. medinalis* were microinjected with 3 µg of dsRNA using glass capillary micropipettes. On the second day post-eclosion, both dsRNA-treated and control females were dissected to examine ovarian development and to collect tissue samples. Transcript levels of *CmVg* and *Cmed074000.1* in these ovarian samples were subsequently quantified.

Quantitative real-time PCR (qRT-PCR) was conducted using a SYBR Green kit (Yeasen Biotechnology, China) following the manufacturer’s protocol. The relative mRNA expression levels of target genes were calculated using the 2^−ΔΔCt^ method (Livak and Schmittgen 2001), with *Actin* and *GAPDH* employed as reference genes for normalization (Zhao et al. 2022, Xie et al. 2025). Three biological replicates were performed, each comprising 20 adult insects. The primer sequences used in this study are listed in Supplementary Table S1; the melting curves and amplification efficiencies for the *Cmed074000.1* and *CmVg* qPCR primers are shown in Supplementary Fig. S1.

### Image Acquisition and Measurement

Ovaries were photographed using a stereo microscope (Mshot, Guangzhou, China) with the following parameters: resolution, 3984 × 2656 pixels; objective, 1.0×; zoom, 1.0×; overall magnification, 1.0×. To minimize measurement error caused by floating ovarioles, ovaries were gently adhered to a wax-coated dish during imaging. The lengths of the first three basal oocytes were measured from the captured images using ImageJ software (Fan 2019, Pudar et al. 2021). All measurements were performed in triplicate.

### 20E Treatments

20E (Selleck, Shanghai, China) was dissolved in anhydrous ethanol and diluted with ddH_2_O to 1, 100, and 500 ng/μL. On day 6 after pupation, female pupae were randomly assigned to three treatment groups receiving intra‑abdominal injections of the corresponding 20E solutions, and a control group receiving an equal volume of vehicle (anhydrous ethanol in ddH_2_O). At 48 h post‑adult emergence, fat bodies were dissected and processed for qRT‑PCR to evaluate the dose‑dependent effects of 20E on *Cmed074000.1* and *CmVg* expression (Gong et al. 2025).

### Data Analysis

The data were analyzed by the one-way ANOVA statistical method using SPSS 16.0. Student’s *t*-test was used for comparison between two groups, and *P *< 0.05 values were considered significantly different. The values were shown as the mean ± SEM for each experimental group.

## Results

### Screening of the Core Active Region of the *CmVg* Promoter

**Fig. 1.**
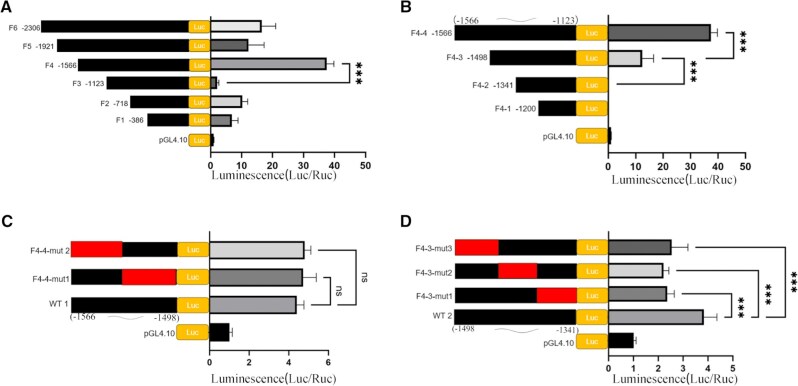
Analysis of promoter activity of the *CmVg* gene. (A) Serial deletion analysis of the *CmVg* promoter revealed that the region from –1566 to 0 exhibited significant promoter activity. (*F* = 110.4, *P *< 0.001). (ANOVA and Duncan’s new multiple range tests). (B) Further deletion analysis of sequences between F4 and F1 showed that the regions from F4 to F4-3 and from F4-3 to F4-2 possessed substantial activity. (*F* = 142.8, *P *< 0.001). (ANOVA and Duncan’s new multiple range tests). (C, D) Mutational analysis of three sub-regions between F4-3 and F4-2 showed significant differences compared to the wild-type, whereas mutation of two segments between F4-4 and F4-3 did not cause significant changes. (*F* = 92.38, *P *< 0.001 and *F* = 62.68, *P *< 0.001, respectively). (ANOVA and Duncan’s new multiple range tests), using the pGL4.10-basic vector as a control.

The putative promoter region of the *CmVg* gene, spanning approximately 2000 bp upstream of the start-codon of translation initiation, was obtained from the NCBI database (Appendix S1). To delineate its functional regions, a series of truncated promoter fragments was constructed and assayed using a dual‑luciferase reporter system. The results showed that the F4 construct’s luciferase activity was significantly higher than that of the F3 construct (Fig. 1A). Based on these findings, the region between F4 and F3 was further characterized, demonstrating that both the F4‑3 and F4‑4 fragments significantly increased promoter activity (Fig. 1B). Subsequent targeted mutagenesis within these regions showed a differential effect: luciferase activity was not significantly altered in the F4‑4 to F4-3 region (Fig. 1C) but was markedly reduced in the F4‑3 to F4‑2 fragment (Fig. 1D). It was indicated that the 158 bp region from “−1498” to “−1341” is the core active region of *CmVg* promoter.

### Identification of BrC Regulatory Elements in the Core Active Region of *CmVg* Promoter

Putative transcription factor (TF) binding sites within the core active region of the promoter of *CmVg* (−1498 to −1340) were predicted using the JASPAR 2024 database. This initial in silico screen identified 44 candidate TFs with high predicted binding affinity. Application of a stringent score threshold (>9.0) filtered these candidates to retain only high-confidence binding sites. TFs with established functional links to vitellogenin synthesis were selected as the primary candidates for subsequent analysis.

**Fig. 2.**
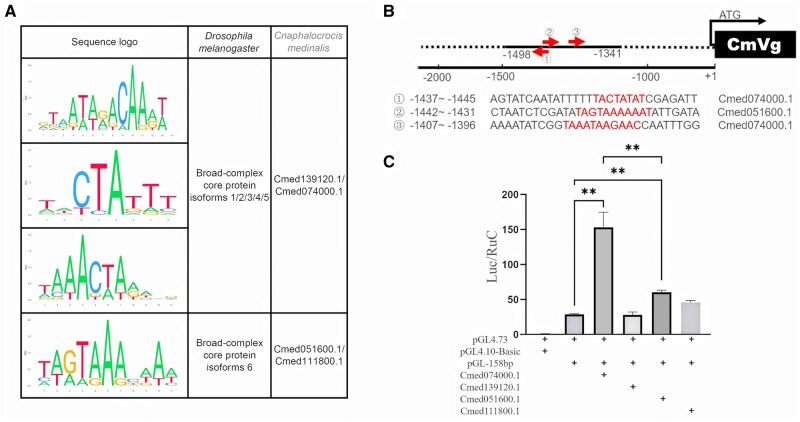
Bioinformatic identification and site‑directed mutagenesis of BrC response elements in the *CmVg* promoter. (A) Prediction of consensus sequences for BrC response elements. Consensus sequences for BrC response elements in *Cmed074000.1*, *Cmed139120.1, Cmed051600.1, and Cmed111800.1* were predicted using the JASPAR 2024 database (https://jaspar.elixir.no/), based on the *Drosophila* BrC response element. The vertical axis indicates nucleotide frequency within the position weight matrix, and the horizontal axis shows the corresponding base position. (B) Predicted BrC response elements in the active region of the *CmVg* promoter. The prediction was performed with the JASPAR 2024database. Upper panel: diagram of the three predicted BrC CREs located in the regulatory region of *CmVg*; lower panel: putative conserved BrC sequences. (C) Effects of four BrC isoforms on pGL‑158bp promoter activity. In dual‑luciferase reporter assays, *Cmed074000.1* and *Cmed051600.1* significantly enhanced promoter activity compared with the control. (*F* = 102.1, *P *< 0.001; ns, not significant). (ANOVA and Duncan’s new multiple range tests), using the pGL4.10-basic vector as a control.

There were four different BrC genes in the genomics database of *C. medinalis* to date (https://www.ncbi.nlm.nih.gov/datasets/genome/GCA_014851415.1/), including *Cmed074000.1*, *Cmed139120.1*, *Cmed051600.1*, and *Cmed111800.1.* All four share a conserved BTB domain (Supplementary Fig. S2). In other insects, distinct BrC isoforms differentially regulate *Vg* transcription—for example, BrC-Z2 activates, while Z1/Z4 repress the *Vg* promoter in *Aegypti*—yet whether any of these four *C. medinalis* BrC homologs participates in *CmVg* regulation remains unknown. Cis-regulatory elements for these four BrC genes were predicted by bioinformatics analysis, revealing distinct profiles for the pairs *Cmed074000.1*/*Cmed139120.1* and *Cmed051600.1*/*Cmed111800.1* (Fig. 2A). According to the JASPAR 2024 database, cis-regulatory elements (CREs) read by *BrC* of *Drosophila* were shown in Fig. 2A. And four homologs of BrC in *C. medinalis* mentioned above were also shown in Fig. 2A. Then, three CREs of BrC were identified, which are located in two loci within the defined core promoter region (Fig. 2B).

### BrC Candidate of *Cmed074000.1* Enhances *CmVg* Promoter Activity

To examine the regulatory effects of various BrC proteins on the *CmVg* promoter, a reporter vector (pGL-158bp) containing the 158-bp core active promoter sequence was co-transfected with pEGFP plasmids expressing distinct *CmBrC* proteins into HEK293T cells for dual‑luciferase assays. Compared with the empty vector control, both proteins of *Cmed074000.1* and *Cmed051600.1* significantly activated the *CmVg* promoter, with a stronger effect observed for *Cmed074000.1* (Fig. 2C). By contrast, *Cmed111800.1* and *Cmed139120.1* produced no significant activation on the 158-bp core active promoter region.

**Fig. 3.**
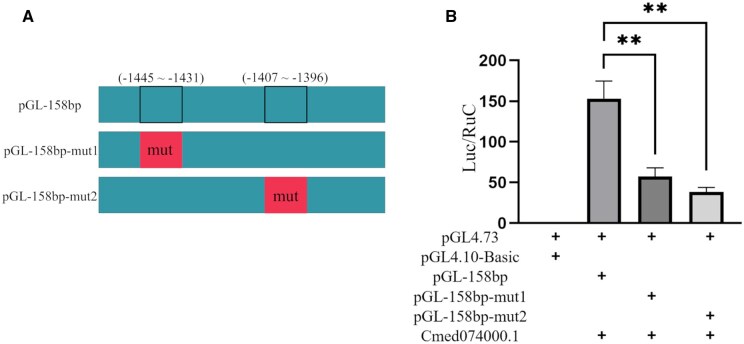
Functional validation of predicted *Cmed074000.1* binding sites in the *CmVg* promoter regulatory region. (A) Sequence alignment of the two predicted *Cmed074000.1* CREs and their corresponding mutants. (B) Effect of site‑directed mutagenesis on *Cmed074000.1*‑induced promoter activation. Mutation of the predicted binding site for *Cmed074000.1* in the pGL‑158bp reporter construct abolished its enhancing activity. (*F* = 82.31, *P *< 0.001). (ANOVA and Duncan’s new multiple range tests), using the pGL4.10‑basic vector as the control.

Given that three CREs (Fig. 2B) and to assess the binding specificity of *Cmed074000.1*, mutant reporter vectors were generated by introducing mutations at two loci within the 158-bp core region (Fig. 3A). Dual‑luciferase assays revealed that mutations at both loci markedly reduced the ability of *Cmed074000.1* to activate the *CmVg* promoter (Fig. 3B).

### Effects of *Cmed074000.1* Knockdown on *Vg* mRNA Expression Level and Ovarian Development

**Fig. 4.**
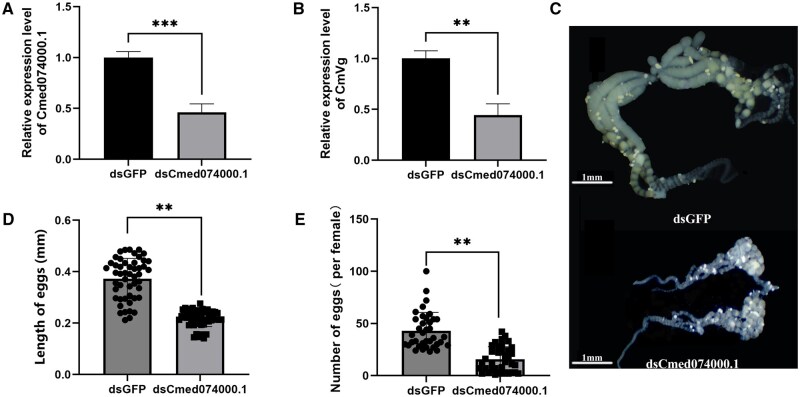
The RNAi-mediated silencing of *Cmed074000.1* impairs reproductive function in female *C. medinalis*. (A) *Cmed074000.1* transcript levels in the fat body 1 day after dsRNA treatment (qRT‑PCR). (B) *CmVg* transcript levels in the fat body 1 day after dsRNA treatment (qRT‑PCR). (C) Ovarian differentiation phenotypes in wild‑type versus dsRNA-treated individuals. (D) Egg-laying capacity of wild‑type and *dsCmed074000.1*-treated females. (E) Lengths of the first three basal oocytes from ovaries of wild‑type and *dsCmed074000.1*-treated females. Data represent mean ± SEM. Between-group comparisons (*dsCmed074000.1* vs. *dsGFP* control) were performed using Student’s *t*-test (**P < 0.01, ***P < 0.001).

RNA interference (RNAi) was performed by microinjecting dsRNA targeting *Cmed074000.1* into female *C. medinali*s, and dsRNA targeting *GFP* served as the control. qPCR analysis confirmed that injection of *dsCmed074000.1* effectively reduced its transcript levels and concurrently downregulated *CmVg* expression (Fig. 4A and B) (Supplementary Table S2 lists the specific Ct values). Compared with *dsGFP*-injected controls, adult females treated with *dsCmed074000.1* exhibited multiple reproductive defects, including impeded development of the ovaries, shorter oocytes, and reduced egg number (Fig. 4C and D). Specifically, egg production decreased by 62.24% in the *dsCmed074000.1* group (Fig. 4E). Together, these results indicate that knockdown of *Cmed074000.1* suppresses *CmVg* transcription, thereby disrupting egg formation.

### Transcriptional Responses to 20E

**Fig. 5.**
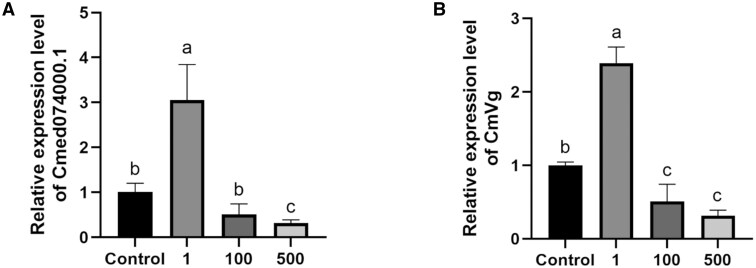
Relative expression of *Cmed074000.1* and *CmVg* after treatment with different concentrations of 20E (1, 100, and 500 ng/μL). (A) *Cmed074000.1* expression after 20E treatment. (B) *CmVg* expression after 20E treatment. Data are presented as mean ± SEM (*n* = 3 independent biological replicates). Different letters above bars indicate significant differences (one-way ANOVA, *P *< 0.05).

To verify the specificity of the RNAi‑mediated knockdown phenotype of *Cmed074000.1*, on day 6 post‑pupation, female pupae were injected intra‑abdominally with different concentrations of 20E; control pupae received an equal volume of vehicle. Fat bodies were dissected at 48 h post‑eclosion and processed for qRT‑PCR (Supplementary Table S3 lists the specific Ct values). Compared with the control group, 1 ng/μL 20E significantly upregulated *Cmed074000.1* and *CmVg* expression by 3.0‑fold and 2.4‑fold, respectively. However, at higher 20E concentrations, the expression of both genes declined progressively (Fig. 5). This upregulation contrasts with the downregulation of *CmVg* observed after *Cmed074000.1* knockdown. These complementary gain‑ and loss‑of‑function results strongly support that *Cmed074000.1* specifically regulates *CmVg* and suggest that elevated 20E levels may suppress this regulatory module via negative feedback.

## Discussion

As a key biomarker for female insect fecundity, *Vg* stands out among reproduction-related proteins (Pereira et al. 2025). Elucidating the function and regulation of *Vg* not only advances our understanding of insect reproduction but also informs the development of targeted pest control strategies. However, while reproductive mechanisms in Lepidoptera have been extensively studied in model species like the silkworm, research on vital agricultural pests such as the rice leaf folder, *C. medinalis*, remains scarce (Deng et al. 2015). To address this, we cloned the promoter region of the *Vg* gene from *C. medinalis* and evaluated its transcriptional activity using a dual‑luciferase reporter assay. A core active region was identified within the promoter. Further investigation demonstrated that the transcription factor BrC significantly enhances *Vg* promoter activity.

During vitellogenesis, *Vg* is primarily synthesized in the fat body, secreted into the hemolymph, and subsequently internalized by developing oocytes via the follicular epithelium. Research over the past decade has established that vitellogenesis is primarily orchestrated by juvenile hormone and ecdysone. This process is further modulated by nutritional signals transduced through neuropeptides and microRNAs (Sun et al. 2002, Park et al. 2006, Hansen et al. 2007). BrC, a key early-response gene in the ecdysone signaling pathway, plays an important role in insect development and metamorphosis (Zhu et al. 2007, Cruz et al. 2012). Moreover, its regulation of *Vg* is isoform-specific. For example, in mosquitoes, BrC-Z2 acts as a transcriptional enhancer, whereas isoforms Z1 and Z4 function as repressors (Zhu et al. 2007). Similarly, in the silkworm, RNAi-mediated knockdown of *BmBrC Z2* impairs ovarian development and reduces vitellogenin deposition (Yang et al. 2014b), and the direct binding of *BmBrC Z2* to the *BmVg* promoter and its transcriptional activation function have been rigorously validated by both EMSA and ChIP assays (Yang et al. 2014a; Lin et al. 2017). Phylogenetic analysis revealed that *Cmed074000.1* clusters closely with *BmBrC Z2* and *BmBrC Z4* (Supplementary Fig. S3). In this study, four BrC genes in the genomics data of *C. medinalis* were analyzed, revealing that only two of them (*Cmed074000.1* and *Cmed051600.1*) influenced *Vg* expression, with *Cmed074000.1* exhibiting the most pronounced effect. Additionally, two potential CREs read by *Cmed074000.1* were identified within the *CmVg* promoter. Transfection assays demonstrated that these two CREs are essential for the regulated expression of *CmVg*. RNAi targeting *Cmed074000.1* resulted in the formation of egg chambers that were visibly paler and smaller than those in controls, suggesting impaired *CmVg* synthesis. In complementary gain‑of‑function experiments, 20‑hydroxyecdysone treatment significantly upregulated both *Cmed074000.1* and *CmVg*, confirming a functional 20E–*Cmed074000.1*–*CmVg* regulatory axis in C. medinalis (Supplementary Fig. S4). Although these functional and expression analyses strongly implicate *Cmed074000.1* in the transcriptional activation of *CmVg*, direct physical interaction between *Cmed074000.1* and the *CmVg* promoter has yet to be experimentally demonstrated. Future EMSA and ChIP assays will be essential to validate the binding of *Cmed074000.1* to the identified CREs within the *CmVg* promoter. Collectively, these findings underscore a significant role for *Cmed074000.1* in vitellogenesis in the rice leaf folder.

Other transcription factors may also regulate *Vg* expression. For instance, Hsc70 acts as a molecular chaperone to negatively regulate *MeVg2* gene expression by binding to the HSF-response element of the *MeVg2* promoter in the shrimp *Metapenaeus ensis* (Chan et al. 2014, Deng et al. 2015). Several findings suggest that the transcriptional regulation of *Vg* is complex and could be influenced by POU (a homeodomain transcription factor) and E74 (Lin et al. 2017, Zhen et al. 2018, Cong et al. 2020). Interestingly, in silico analysis of the 158 bp core active region of *CmVg* promoter also predicted a binding site for the POU (*CmSGF3*) near the Cmed074000.1 CREs (Supplementary Fig. S5A). Dual-luciferase reporter assays demonstrated that *CmSGF3* significantly inhibited the activity of the 158 bp core active region of *CmVg* promoter (Supplementary Fig. S5B). It was reported that POU can interact with BrC and inhibit its transcriptional activity in *B. mori* (Lin et al. 2017, Cong et al. 2020). Thus, we will delve into the relationship between protein products of *Cmed074000.1* and *CmSGF3*, and their impact on *Vg* transcription in the future. In addition, are there any other regions in the *CmVg* promoter that serve as BrC recognition sites, besides the 158 bp core active region? The results of the bioinformatics analysis using the JASPAR database support this idea. Dual-luciferase assays performed on the F4 fragment (−1566 to −1) showed different states of expression activity regulation, compared to that of the 158 bp fragment (−1498 to −1341), especially the difference between *Cmed074000.1* and *Cmed051600.1* (Fig. 5C and Supplementary Fig. S5C). It indicated that the regulation of *CmVg* by BrC family members is complex, and we cannot limit our eyes to the 158 bp core active region.

From an applied standpoint, identification of the *Cmed074000.1*–*CmVg* regulatory axis opens promising avenues for pest management. The 62.24% reduction in egg production following *Cmed074000.1* knockdown confirms that even partial disruption of this transcriptional node can markedly compromise reproductive output. With the growing feasibility of RNAi-based pest control via transgenic crops or sprayable dsRNA formulations, *Cmed074000.1* constitutes an attractive molecular target for suppressing populations of this migratory rice pest (Mateos Fernandez et al. 2022). Furthermore, because the ecdysone signaling cascade is restricted to arthropods, targeting BrC-mediated reproductive pathways may provide a favorable ecotoxicological profile with minimal risk to vertebrates or beneficial organisms. Future efforts should prioritize evaluating dsRNA sequence specificity toward non-target lepidopterans and developing efficient field delivery platforms. Ultimately, integrating BrC-targeted reproductive interference with existing cultural and biological control practices could contribute to more sustainable integrated pest management of *C. medinalis* in rice agroecosystems.

In summary, this study establishes *Cmed074000.1* as a critical transcriptional regulator of *CmVg* and female fecundity in *C. medinalis*, based on a synergistic combination of in vitro promoter assays, in vivo RNAi knockdown phenotypes, and complementary 20E gain-of-function experiments. By uncovering this key reproductive pathway, the work not only advances fundamental knowledge of reproductive biology in this economically important pest but also highlights a concrete molecular target for developing novel, environmentally sound management strategies.

## Supplementary Material

ieag057_Supplementary_Data
