# Supplementary material for: Identification of the core region responsible for the activity of CmVg promoter and its regulatory transcription factor BrC in Cnaphalocrocis medinalis (Lepidoptera: Pyralidae)
Source: J Insect Sci. 2026 Jul 21;26(4):ieag057. doi: 10.1093/jisesa/ieag057 (PMC13386126; doi:10.1093/jisesa/ieag057)
Supplement: ieag057_Supplementary_Data [file ieag057_supplementary_data.zip › Supplementary_File _with legends.docx]

**Fig S1**. Melting curves and amplification efficiencies of qPCR primers for *Cmed074000.1* and *CmVg*. (A) Melting curve and (B) amplification efficiency of *Cmed074000.1*. (C) Melting curve and (D) amplification efficiency of *CmVg*.

**Fig S2** Schematic representation of the protein domains of four *BrC* subtypes in the rice leaf folder, *C. medinalis*. Subtypes: *Cmed074000.1, Cmed139120.1, Cmed051600.1, Cmed111800.1.*

**Fig S3** Phylogenetic analysis of BrC proteins from the rice leaf folder (*C. medinalis*) and other insect species.

A maximum likelihood tree was constructed based on the amino acid sequences of BrC using MEGA X with the JTT+G substitution model. Bootstrap support values (1,000 replicates) are indicated at the nodes; only values ≥50% are shown. The scale bar represents the number of amino acid substitutions per site. *C. medinalis* sequences are shown in bold red. The analysis included BrC sequences from 18 insect species: *Ostrinia furnaca*lis, *Chilo suppressalis*, *Bombyx mori*, *Trichoplusia ni*, *Spodoptera frugiperda*, *Helicoverpa armigera*, *Manduca sexta* (Lepidoptera); *Apolygus lucorum*, *Nilaparvata lugens*, *Bemisia tabaci* (Hemiptera); *Aedes albopictus* (Diptera); *Tenebrio molitor*, *Anthonomus grandis* (Coleoptera); *Dufourea novaeangliae* and *Athalia rosae* (Hymenoptera). GenBank accession numbers are listed in the figure.

**Fig S4** Regulatory mechanism of the BrC transcription factor (*Cmed074000.1*) in mediating 20-hydroxyecdysone (20E)-induced *CmVg* gene expression in the rice leaf folder, *C. medinalis*.

**Fig S5** Dual regulation of *CmVg* promoter activity by *CmSGF3*. (A) Predicted SGF3 response elements in the active region of the *CmVg* promoter. The prediction was performed with the JASPAR 2024 database. Upper panel: diagram of predicted SGF3 CREs located in the regulatory region of *CmVg*; Lower panel: putative conserved SGF3 sequences. (B) Regulation of promoter activity by *CmSGF3* alone or in combination with *CmBrC* isoform: co-expression leads to synergistic activation, while *CmSGF3* alone exerts an inhibitory effect. Data represent mean ± SEM. (C) Effects of four BrC isoforms on *CmVg* promoter activity. (F = 101.7, *P* < 0.001). (ANOVA and Duncan's new multiple range tests).

**Table S1** Primer information

**Table S2** RNAi Ct information

**Table S3** 20E Ct information


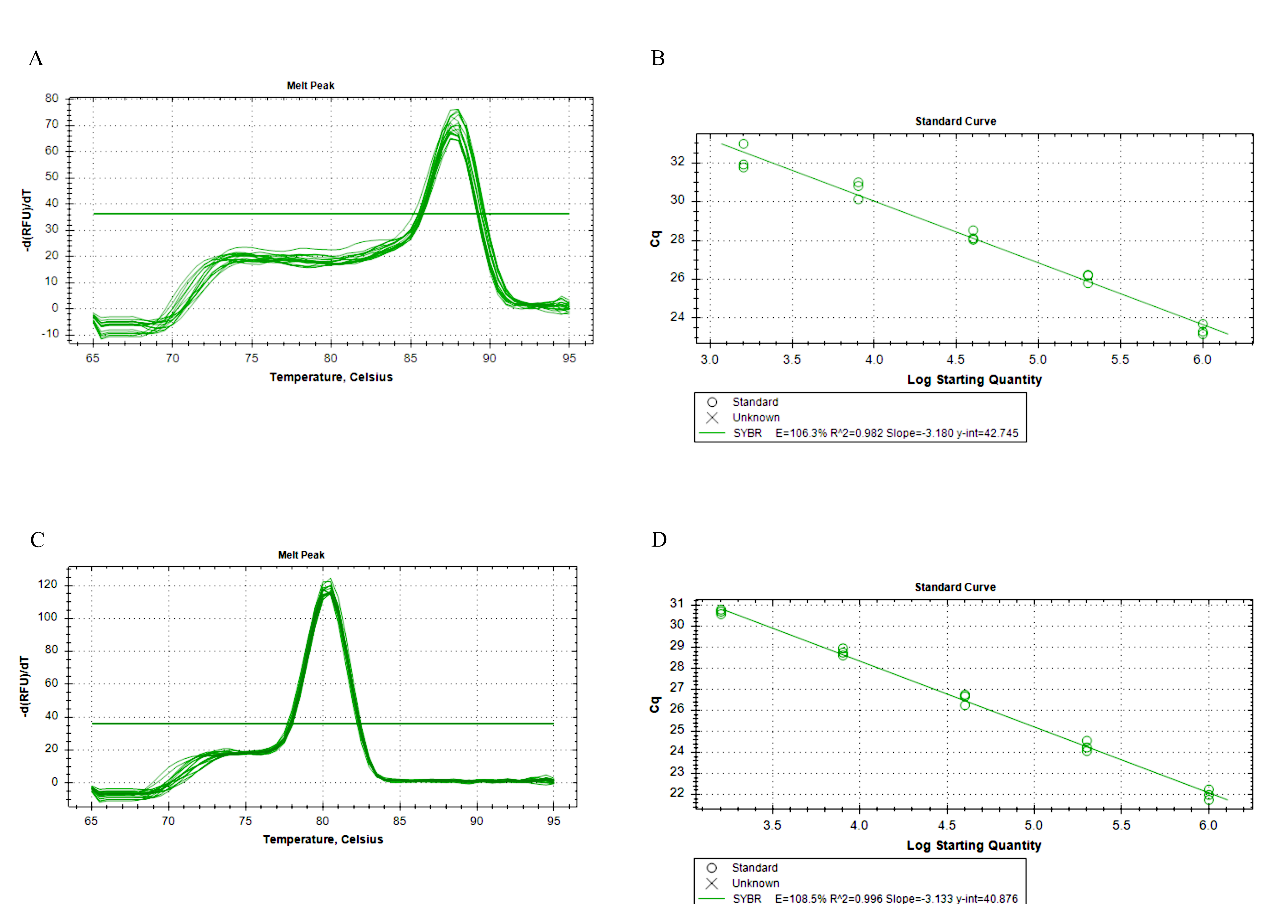


Fig S1. Melting curves and amplification efficiencies of qPCR primers for *Cmed074000.1* and *CmVg*. (A) Melting curve and (B) amplification efficiency of *Cmed074000.1*. (C) Melting curve and (D) amplification efficiency of *CmVg*.


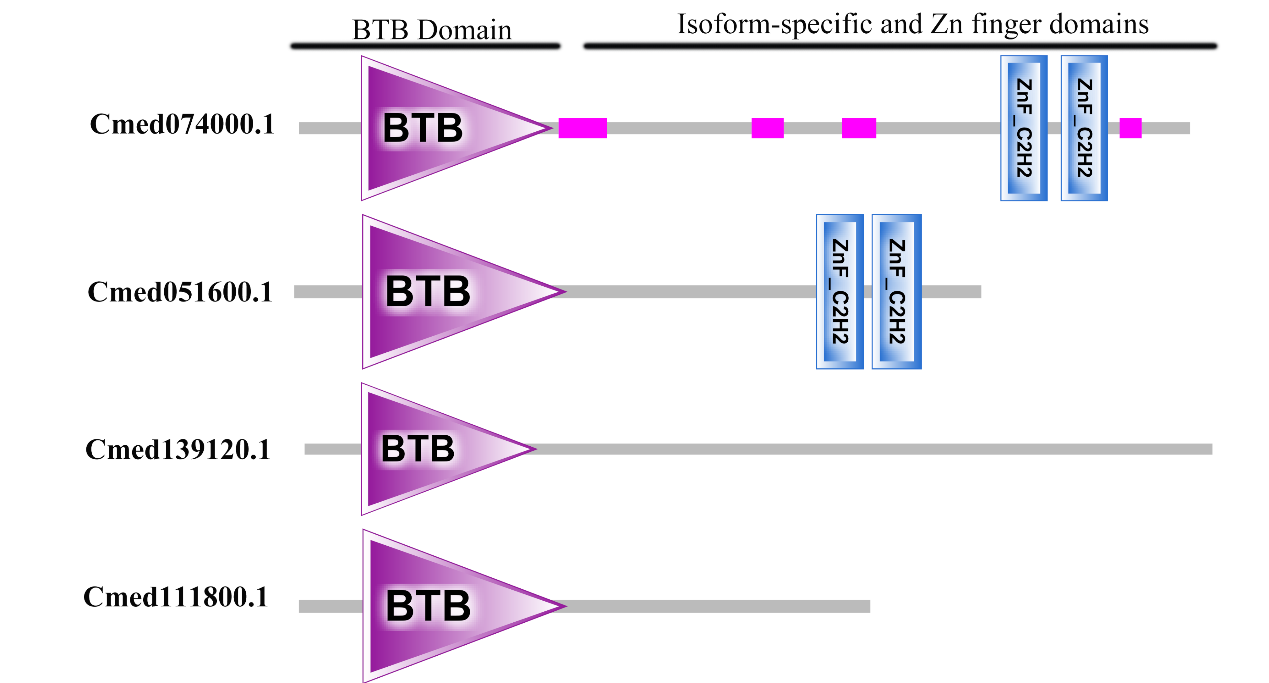


Fig S2 Schematic representation of the protein domains of four BrC subtypes in the rice leaf folder, *C. medinalis*. Subtypes: *Cmed074000.1, Cmed139120.1, Cmed051600.1, Cmed111800.1.*


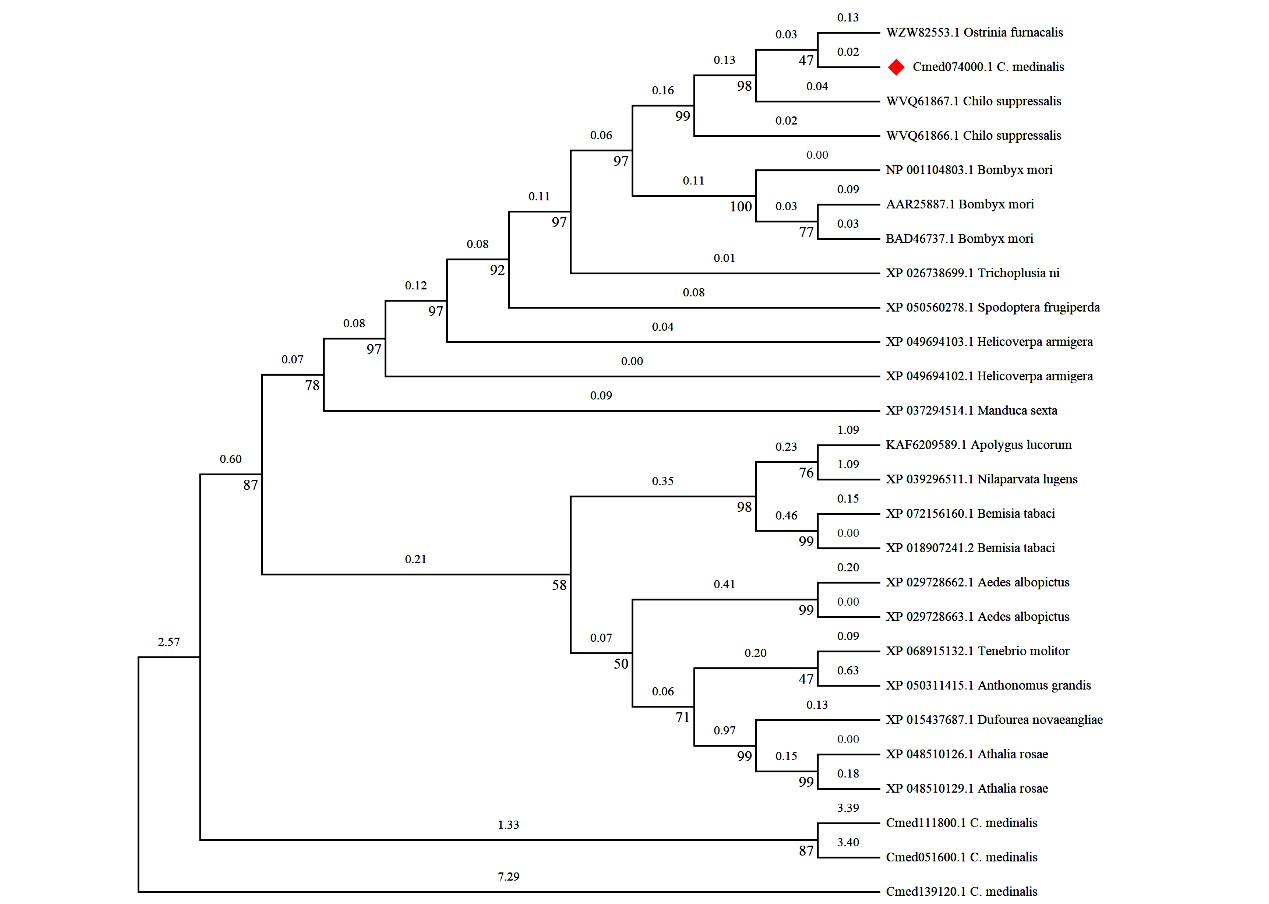


Fig S3 Phylogenetic analysis of BrC proteins from the rice leaf folder (*C. medinalis*) and other insect species.

A maximum likelihood tree was constructed based on the amino acid sequences of BrC using MEGA X with the JTT+G substitution model. Bootstrap support values (1,000 replicates) are indicated at the nodes; only values ≥50% are shown. The scale bar represents the number of amino acid substitutions per site. *C. medinalis* sequences are shown in bold red. The analysis included BrC sequences from 18 insect species: *Ostrinia furnacalis, Chilo suppressalis, Bombyx mori, Trichoplusia ni, Spodoptera frugiperda, Helicoverpa armigera, Manduca sexta* (Lepidoptera); *Apolygus lucorum, Nilaparvata lugens, Bemisia tabaci* (Hemiptera); *Aedes albopictus* (Diptera); *Tenebrio molitor, Anthonomus grandis* (Coleoptera); *Dufourea novaeangliae and Athalia rosae* (Hymenoptera). GenBank accession numbers are listed in the figure.


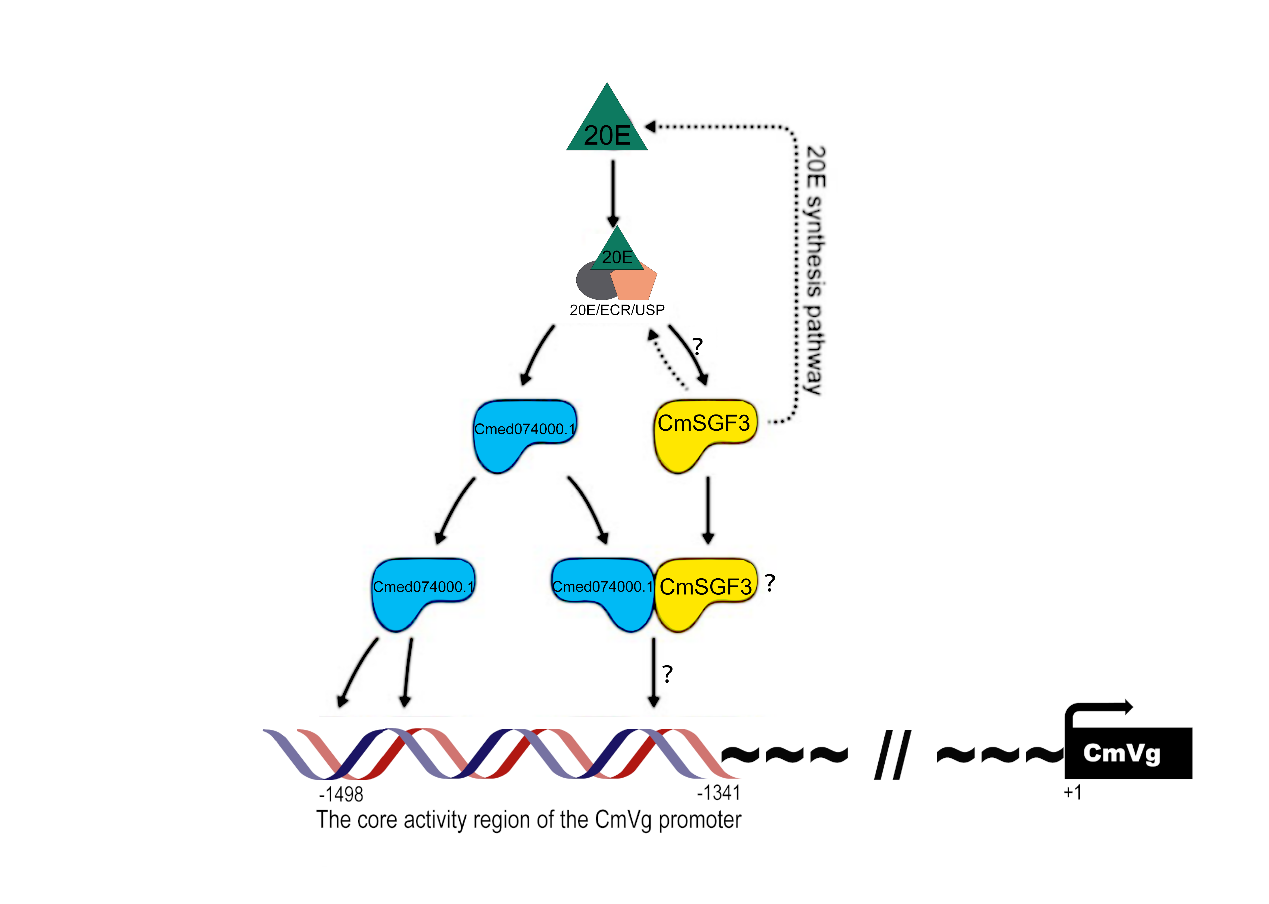


Fig S4 Regulatory mechanism of the BrC transcription factor (*Cmed074000.1*) in mediating 20-hydroxyecdysone (20E)-induced *CmVg* gene expression in the rice leaf folder, *C. medinalis.*


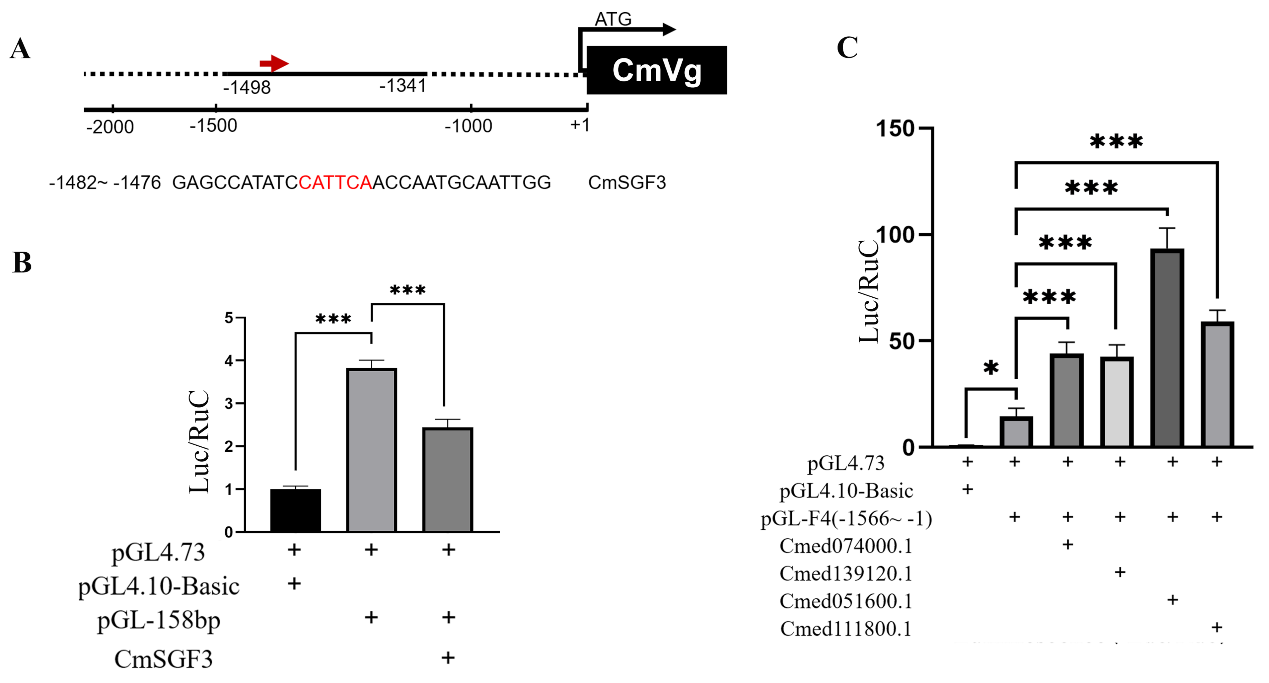


Fig S5 Dual regulation of *CmVg* promoter activity by CmSGF3. (A) Predicted SGF3 response elements in the active region of the *CmVg* promoter. The prediction was performed with the JASPAR 2024 database. Upper panel: diagram of predicted SGF3 CREs located in the regulatory region of *CmVg*; Lower panel: putative conserved SGF3 sequences. (B) Regulation of promoter activity by CmSGF3 alone or in combination with *CmBrC* isoform: co-expression leads to synergistic activation, while CmSGF3 alone exerts an inhibitory effect. Data represent mean ± SEM. (C) Effects of four BrC isoforms on *CmVg* promoter activity. (F = 101.7, P < 0.001). (ANOVA and Duncan's new multiple range tests).

| Table S1 Primer information | | |
| --- | --- | --- |
| Primer names | Sequences (5′-3′) | Used for |
| R | cagtaccggattgccaagcttTTTTGACTCGTTTCGTTGTC | Promoter-pGL4.10 constructs |
| F1 | cctgagctcgctagcctcgagGCGCGATGTAATTGGACAC |  |
| F2 | cctgagctcgctagcctcgagAAGAGCTATATGGTGGTGGC |  |
| F3 | cctgagctcgctagcctcgagGCTTGCCCAAAATATGTGTTCT |  |
| F4 | cctgagctcgctagcctcgagCTTAGGACGCAATCGGGAAT |  |
| F5 | cctgagctcgctagcctcgagGGGGGAAAGGGGTTAAAAGT |  |
| F6 | cctgagctcgctagcctcgagACGTTACTGCCATTGTATTGC |  |
| *Cmed111800.1*-F | ctaccggactcagatctcgagATGGCAGATCAGTTTTGTTTACGTTG | Promoter-pEGFP constructs |
| *Cmed111800.1*-R | atggtggcgaccggtggatcccgTCAGTCATTTTCTGAGTTAACAATTTGAGG |  |
| *Cmed051600.1*-F | ctaccggactcagatctcgagATGAATAATGCACCGCAATTTTCCT |  |
| *Cmed051600.1*-R | atggtggcgaccggtggatcccgCTAAATATGATGAGTGAATGGCTTTGGAC |  |
| *Cmed139120.1*-F | ctaccggactcagatctcgagATGTTAGAACGAAAATATAGTTTACGTTGGGAT |  |
| *Cmed139120.1*-R | atggtggcgaccggtggatcccgTTAAAATTTTTGAATCATGAAAGTCATGTTGGTGC |  |
| Cmed074000.1-F | ctaccggactcagatctcgagATGGTGGACACACAGCAC |  |
| *Cmed074000.1*-R | atggtggcgaccggtggatcccgTTAGACGTTGAATTGATCTTTGA |  |
| *dsCmed074000.1-*F | gcgtaatacgactcactataggtCACTCCCTGGTCAACAACA | In Vitro dsRNA Synthesis and qRT‑PCR Analysis |
| *dsCmed074000.1-*R | gcgtaatacgactcactataggtGCCATGCTGAAGTGGTAAG |  |
| *QCmed074000.1*-F | CCTTCGAGAACCTTCGGGAC |  |
| *QCmed074000.1*-R | GGTGCTTGCATGGTGTTGAC |  |
| *Actin*-F | ATGGTCGGCATGGGACAG |  |
| *Actin*-R | GAGTTCATTGTAGAAGGTGT |  |
| *GAPDH*-F | CTGCCACTCAAAAGACCGT |  |
| *GAPDH*-R | AAGGCCATACCAGTCAGT |  |

Table S2 RNAi Ct information

|  | *dsCmed074000.1* | *dsCmVg* | *GAPDH* | *Actin* |
| --- | --- | --- | --- | --- |
| Control | 19.28 | 19.39 | 16.47 | 25.13 |
|  | 19.57 | 19.77 | 16.22 | 25.08 |
|  | 18.98 | 19.42 | 16.17 | 24.61 |
|  | 19.09 | 19.25 | 16.20 | 24.79 |
|  | 19.38 | 19.53 | 16.70 | 25.40 |
|  | 19.62 | 19.75 | 16.58 | 25.06 |
| Treated | 20.08 | 20.44 | 16.38 | 24.99 |
|  | 20.35 | 19.95 | 16.45 | 25.06 |
|  | 20.12 | 19.99 | 15.85 | 24.69 |
|  | 19.74 | 19.69 | 15.59 | 24.73 |
|  | 19.73 | 20.14 | 15.02 | 24.83 |
|  | 19.80 | 20.21 | 15.04 | 24.75 |

Table S3 20E Ct information

|  | *Actin* | *GAPDH* | 1 ng/μL | 100 ng/μL | 500 ng/μL |
| --- | --- | --- | --- | --- | --- |
| Control | 25.18 | 16.99 | 21.28 | 20.47 | 21.38 |
|  | 25.09 | 17.06 | 21.09 | 20.19 | 21.31 |
|  | 24.46 | 16.69 | 21.18 | 20.16 | 20.72 |
|  | 24.26 | 16.73 | 21.19 | 20.30 | 20.73 |
|  | 24.67 | 16.83 | 21.27 | 20.28 | 21.16 |
|  | 24.74 | 16.75 | 21.21 | 20.30 | 21.15 |
| Treated | 24.74 | 16.32 | 18.80 | 21.09 | 22.54 |
|  | 24.30 | 16.18 | 18.76 | 21.18 | 22.59 |
|  | 23.41 | 16.28 | 19.03 | 20.92 | 21.35 |
|  | 23.57 | 16.28 | 19.13 | 20.95 | 21.74 |
|  | 24.04 | 16.41 | 19.59 | 20.31 | 22.55 |
|  | 24.02 | 17.37 | 19.31 | 20.45 | 22.57 |
