# Supplementary material for: Identification of the core region responsible for the activity of CmVg promoter and its regulatory transcription factor BrC in Cnaphalocrocis medinalis (Lepidoptera: Pyralidae)
Source: J Insect Sci. 2026 Jul 21;26(4):ieag057. doi: 10.1093/jisesa/ieag057 (PMC13386126; doi:10.1093/jisesa/ieag057)
Supplement: ieag057_Supplementary_Data [file ieag057_supplementary_data.zip › Appendix 1.docx]

>**CmVg promoter sequence**

ACGTTACTGCCATTGTATTGCAACTTATGTAAAAAGTACTAATAAATAATTTAATTATAGTTATAGACTTAAATACTCTCATTTAAGATGAGATGCAATTTGTACAAATGCTTGCTTTCCAAAGAATCTCCTAATATTAAGTATTTTCCAAAATAAGATCTCTTAAATAGGAGTGCCTAATGGCAGTTAAGTTTAAAGGCAGGAGCTGCCGTTAAATTTTAATATTCAGTTAAGAAGTTTGCTCAATTTTTATTGATATTAAAATTTTAGTTCACGGAATATGAAATCAAAACGCATTTTTATTTTTAACCTTTCTATCTTCAACCCAATGCAAATAATACATGTACCTGCACTTTTATCGATAGTAATGAAAAAAAAACATTTGGGGGGAAAGGGGTTAAAAGTCGGCAGCGCACTTGTAGGTACTCTACTGGTGTTGCAGGTGTATCATATCGCTTACCATCAGGTGATCCGTCTACCCGTCTGCCATTTTAATTTAAGTATTTTTATTTTAAGCACCTAGCTTGTAAAAAATCTGATTTGCCCCTACTGGCCCAAAACCTGGGTACGCCCATGGGCACCACTGCTTGACAAAAATTGTACTTGATATAATGGGGAATAAACGAGAGGATATTTAAAATAATACATTAAGTAGGTATTTATGCTTTCTGCCTTGCCCAATGGCTATGAGATTATGGTCAATCATAGTGTTATGGTACCACCCGGGCTTCAAGGGACAGCTTAGGACGCAATCGGGAATTCCCCGTCTTCAGGGCAAAGACTTCCCTACTCATTTTCCAAACATTTTCGTCACGAGCCATATCCATTCAACCAATGCAATTGGGTGTTTGACTAATCTCGATATAGTAAAAAATATTGATACTCTATCAAAATATCGGTAAATAAGAACCAATTTGGCCAACATTTTCAGTTAAAAATATAACGTCTACTTAGTTGGTTTTGTGAGCATTCAAAGTTTCATGGGCAAGTCCATTTTTAAAGTTATAAAATTTTAAGAAAATAATTATATCATTGAACTACCCGTACATTTTCCACCAACTTTTATTATAAAACCATTTACTTAAGTACTTAAACGAAACCAAACAAAATTTACTAGACGCGCACACAGAAATTTGAGTTGTGACATCACAATTCAATAGCCATTTGTATAGAGCGTTTTTGGGCTTGCCCAAAATATGTGTTCTTAAAGTTGTGATATCTCAGGTAATTCAAATGAGGATCGAGTAATTCTTTCAACGATGTTTCATAAATTGAGACGTCATTTGATTAAACACATAAAAAATTTTAGTCAAATACCCAATTTACCGTGGGTTTTTATTTAGGCTTCGGCATAAACGGACCGGCTCGACCAGAGTGATACCACGGCCTCGCATAGGACCGGCGTGAAGCAGCGCATGCGCTGTGTTTCGTCGAGTGAGTGAGGATACTAGAGAGCCTATTGGGGGGAACTGTGGTTAGGTTAGCAGCGCACTTGTGGCTCTACTGGGGATGCAGGTGTCTATGGATGGCGGCAATCGCTTACCATCATGACATGATCTGTCTGCTTGTCTGCCATCTATCACATAAAAAGAGCTATATGGTGGTGGCGCTTCTTTGGTCTAGGTTTTTAAGTACTGGGCCATAAATAATCTAGGGGTCCCGGGTTCGATTCTCAGGTCGAGCAAAAGTTAATAATATGTTTTGAATTAATAGCTCGTGTTTGTGATCACCAATCCAAATTAGGCGAACGTGGTAATTAAATCTAATCCCATCATAGCGATGGCTTCATTTCTATGATGGAGGCCTTTGCCCTGCAGTGGGACAGTATATATCTTCTTCTATCTTCTATACTATTTATAAAGAGATAACTTCTATAGACAACGACAAGGCTTTTAGGTAAACCGAAAACACGAAACGCTTGCGCGATGTAATTGGACACAGCGCCATATATCGGTTCATTTCTGAATTACGGTCTTGAAGTGAAGTGAGGGCGGGCCACTATAGGCTGATACTTTGCTTTTTATATTATGGTGGGTTTATTTACCTATACAGACAACATAGAAATACGCAGCTAACTATTATGTACGCAAAAGTGACATCATGATTAAAATCTCCGATTAGTTACCTCGTGCCAATCTAAGTCTTAGAATTAGAAAACACGCAACATTGTATGTTATCACGGGTCAAAGTTAGTTCTTCCACCCCTAGCTCGTGTATGTTTGATCATATAAAAGGGGGGGTTCGGCCTGTAAGCAGCAGAAGTCACCTTCTCGTTAAACCACTCAGGACAACGAAACGAGTCAAAA**ATG**AAGATCTTGGTATCGGCTGCCCTTATTGCGGCCGTAACATCTAGCCACCGAAGCAGCCAAGATGTCGACAACCAGACATACGGACCCTGGCGAGTGGGGACCTCCTACCGGTACGACGTGGACTCCTTCACGGTAGCCCGGCTGCATGAGGGCCAGAGCAGCGGAACTGCCTTCAAGGCCCACTTCGTGGTCCGTGTTCAGGCCGACGGACGCCTCCTCGCGCGCCTCGAGAACCCTCGCCACGCTCAGGTCCACCAAGAGCTTCCGGGCGACCGCCAGCTGCCCTCCGACCTCAAGTACCAGCCCCTCGACAAACTCGACCAGCCCTTCGAGATCCTCGTCGACGGCGGCCGCGTCGTCTCCCTCGAACTCCCCGCGAACCTGCCTCTGGCCCACGAGAACTTACTGAAGGGCCTCATTGGCTCCCTCCAAGTCGACCTCTCCACCTACAGGCATGTCCGCAGACAGGAAAACGGATTCGACCAGCAAACTCAACAGGGTATGTTCAAGAAAATGGAAACAGACGTCACCGGCGACTGTGAGACCATGTACAACGTCTACCCAGCCATCCCTGAGTACCGCCGCGAGCTGCCCAACTTCGTGAACGATGAAGAACCCATCATGATCTCCAAGACCAAGAACTACGGACATTGCCACCACCGCGTCGCCTACCACTTCGGAGTACCCCAGGGCTCTGAATGGTCCGGCACCGCGCACAAGAACCATGAGGAGCAGTTCATCAGGCGAGCGACGGTTTCCCGCATCCTGTCTGGCAAGCAAGGCCCTATCTATAAGACTGAGACCACCAGCACCGTTCATGTAAACCCTCAAATCTATGGCAAGGATCAGGCTGAAGTACTCAGCTACGTCCAACTGCAGTTGGTATCATACGAGCAGGACAGCCAGCCTGAATGGCAGAAGCCTGAACACTACCGCCAGGTCAAGAACCTTTTATACTCCGTCTCGCAGAAACAGATCGATATCCAAGATTGGTCATCATCGGCGGAGTTGAACGAAGACAAGTACCAAGACAATCAGGTTTCCGAAAACGAACAGTACGGTCAGGATAATGAACAGCAACCTGAAGAAGCTATCAACCGTATGCGCCGTTCAGCTAAGGCATCTAAAGAAGATTCGATGTACAGATACCAGCAATTCGAGGCTGCTAGCTCTTCACAGTCCGCCAGCGACACCGACTCAGCTTCGACCTTCGTTAATGAAGACATCCCAAAAAACAACGAGCCTGCTTACGCCGCTTTGTACATGACCCCCCAGTCATCCGCCGAGAAGAAGCAGAACCCAGTGAACGCTCAGAAGCTTGTGCAGGAGTTGGCTCAGCAACTGCAGAACCCTAACAACATGCCCAAAGGAGACTTCCTGTCCAAATTCAACGTCTTGGTCCGCATCATCGCTTCCATGAGCTCGGAACAACTTACCCTCACAAGCCGCAGCATCGAAGTCGCCAAGTCTTCCACAAACAAAGTCAAGTCTGACATGTGGATGATCTACCGCGATGCCGTCACCGAGGCCGGTACCATGCCCGCGTTCCAGCAGATCAAGACCTGGATTCAGACCAAAAAGATTCAGGAGGAGGAGGCCGCTCAGGTTGTCGCTTCCCTGACGCACACCCTTCGTTATCCCACCAAGGACATCATGATCCAATTCTTCAAGCTCGCTATCAGTGACGAAGTCAGACAGCAGAAGTACCTTAACAGCACCGCTCTGATCTCTGCCACCAGATTCATGAACATGGGTCAGGTCAACAACGAGACCGCTCACTCTTACTACCCCACCCACATGTACGGCCGTCTGTCTCGTCAGAATGACGCTTTCGTCCCTGAGTACGTCCTGCGCCGTCTGTCCGAGTTCCTGAAGCAAGCTATCCTAGAAGGCGACAGCAACAAAGCCCAGGTGTACATTAAGGCTATTGGAAACCTAGGTCACCGTCGTATTCTCAATGTCTTTGCTCCGTACCTCGAGGGTAAGATCGCAGCGTCGACATACCTTCGCGCACAGATGGTGAAGAACCTCCAAGTCCTTGCCCACCAGCAGGATACCGAGACTAGAGCTGTTCTGTTCTCCATCCTGAAGAACATCGCTGAGCCCTATGAAGTGCGTGTGGCTGCTATCGAGAACATCTTCATGGCTCGCCCCACCACCTCCATGATGCAGGCTATGGCCCAGATGACCCGCAACGACCCCAGCGTGCAAGTGCGTGCTGTGCTGAAGTCTAGCATTGAGTCCGCCGCTGAGCTGAAGAACCCCCACTACCTGGATCTGGCTAGGACCGCCCAAGCCGCCAAGTGGATGCTCACCAAGCAAGACTACGGAATCCAATACTCCAACAAGTACCTGAACGAGCACAGCGACCTTGAGACCGAGATCAGCATGCTGAGCGCCTTCTACCACATGGGAAGCGATGACAGCCTTTTCCCCAAATCCCTGAGGTACTCCATCAAGAGCAAGGCTGAGGGCTGGGACAAAGAACAGACGATTTCCACTTCCTTCTCCAGTGTGCAGCGGTTCGTCTACGCTTTCCAGCAGCAAATGAACAGAATGAACAAACAGAGCCCCAAGTCTGAGGCCCAGCACAAGTACTCCGCCCAGAAGGTCTCCGAGATGCTGAACATTAAGCCTGAGCCCCAGGACCCTCTGGAAGCTGCCTTACTCGTGGCCATTATGGGCCAAGAGAGGTACTTCACCTTCAGCGAGAACGACCTCCAGCAACTGCCCCTCACTATCGGCCAGTTCTTCTCCAGCCTGACCAAGGGCGCTGAGAGCCACTACACCAAAGTCCTGAACCAGGCCCAAGTTTCCATCATGTTCCCCGTTGGCATGGGCATGCCTTTCATCTACAAATACAAGACACCCACCGTAGTCCACTTCCAGGGCAAAGTGCAGGGTCAAATCAACCCTCAATCCAAGGACGCTGCTGATATCAATGGAGAAATGCAATTCACGTTCGCAAGGAACATTGACGGCAGTGTCGGTTTCATGGACACGCTCTCCAACCAGTTTGCCAGCGTTGGTGTGATCAGCAAATTCCAACTGAACGTACCCCTCAAGATGCAACTGCAGCTGAAGGCTGGCGACCTTAAGATCCAGATGGAGCCCCTCCGCGCTGACCAAGACAACACCATCGTCCACTACAGCGTCTGGCCTTACTCCGCCTACCAGAAGAAGGACTCTCTTGTCCCTGTTGCCCTCGACAGCGCCACCAAAGTTACCTCCCGCAAGAACAAGGTCGTTTCCATCGACACCAAGTTCGGACAGGCCATTGGCAACCAGTTCCAACTGCAAGGATACTCCCACTCATCTGACTACAAGAGCCTTGGCAACTTGTTGTCCCAGGACTTGTTCACAACAATCAACAATGCCTTCTACCAGAGGGATGTGGCGTTGACCCACTTCAACTTCAAGTATCTTGGAAAACAAAGCCCCAACAACCGCATTACCTTGACGGCTGCAATTGACACATACTACAACCAGCAATACCCCGCTGAACCCCTCGTCCCGACCAACGTTGCTGAAGTTGGACCTAACAACGAAGCCCGTCGCCAGCAAATAGTGAAGCGCGTCGCCGCTGGCATCAACTCCGCCAGGGTCCAAGTCCTAGACGTGAGCGCCACCTACGAAGGAAAACAGAAACACGAATACGTACTGACCGCCGCCATTGGTGACAGCAAGGTTGACAACAAGATCCACTACGCTTTCTTCGCTGGCAGGAACTCCGCCCAACTTGGTGACTCCCAGGTCAACGGTGTAGCTACAGTCAACAAGCCCGAGGTCACTTCTCTCAACTTCCTCGAGGCTCTGAAAAAGAATCTGAAGATGAACTTCGAAGCTGAGATCAAATACGGTCAGAACGGACAGATCCGCATGACTGGCCACACTGAGCGCACTCAGAAGTACGCCGAAGAGCTGAAGAACCACCCTGAGGGCAAACAGTGCGCCCAAGAGATTGAAAAGAACAACTTCTACCAGATCGCTTGCCAGAGGATGATTGTAAGAGCACACGCTCCCGACTACATGAAGGCATCAGTTATCTACAAGGATGCCAGCCCAGTTTTGAAGAGCATGACTTACAAGGCCGTGAGGATCGCCGAATACCTCGGTTTCTGGTACTCTGATGTCAACGCATTGAAGGTTCAGCCCGATGGAAAGTTCGAAATTGAATCGCAGATGAACTACCTGGATGACTCCATGAACATGGTCCTTGGGTCCAGATACGGCGAGCTCCGCTTCAGCAGCGTGCCGATCCCGAAGGTGACTCCGTTCGCCCTCGCTGTCTACTCTCCCATGCAACCTATGGAACGTGTCTTCAACTTCTACACCCGTCAGCAGTACCAACCCTCCTGCACCGTCGACGGCCGCAAGATCAGGACCTTCAGCAACCGAAGCTACGACTACACCCTGACCCGCAACTGGCACGTGGTGATGCAAGACGACGGAAACAGGCTCGGCCGCGACCAGGAAAGTGAGCAGCTGGTGGTTCTGGCCAGGAGGCCGAACGAGAAACAGCAGGAGATCTACATCTCTTACAAGTCTGGAAGCAAGGACTTGGAACTGGAGATCCAGCCCGCCCAGCCCAACGGCAGACCCACCCTGAAGGTGAAAACCAACGCCCAGAAGGTTGTTCAAGGAGACCTGACCATCTACTGGAACTCTGCTGATGGTCAGCCTCTGCTGCAGTACTACACTCGCCCTGATGGTGTTCTGGTGGTTGACATTGAGGAAGGCCGCCTCCGGGCCATGTACGATGGCGACAGGCTGGTTGTCCTAGCTAGCGAGAACAGGAACAACATCCGTGGGATCTGTGGCTACATGAACGGCGAAGCTCGTTACGACTACCTCTCTCCCAAAGGCCTCCTCGACCGCCCCGAACACTACGGTGCCTCCTACGCCTTAAACGACGGAGAGACCGACGAGAAGACCAAGCAGTGGCAAGAGGAAGCCCAGAAGTACGCATACCCTACGAAGAGCCACTACACCGCTATCCTCCCCGCTGACCAGTACTGGAGGAAGGCTAGACAAGTCTCTGAGGAGCACAAAGAAAAGTCGTACCTTAACGTGTACTGGCCAAGGAGCTACAAGAAGTCCCATGGCGAATGCAAGATCCACCAACAGATCCAGTATTTCGAGGACCACAGTGAGATCTGCGTGACGACTGAGCCTGTTCCAGCTTGCGAGTCGAACTGCAGGGGAGAAGGTCACCAGGTCAAGGACGTGCAGGTCGTGTGCAGGCCTAAGGTCGACCAGCAGTTCAAGAACTACAGGAACCAGATCCGTCACGGAGAGAACCCTCAGGTGACTGGTGCTGCTCAGAAGACTAGGCAATACAGGGTACCTAACTCTTGCAGAGCG**TAA**
